# α-Tocopherol Stereoisomer Profiles in Matched Human Maternal and Umbilical Cord Plasma

**DOI:** 10.1093/cdn/nzab073

**Published:** 2021-05-03

**Authors:** Matthew J Kuchan, Stephen J DeMichele, Karen J Schimpf, Xinhua Chen

**Affiliations:** Abbott Nutrition, Discovery Research, Columbus, OH, USA; Retired from Abbott Nutrition, Discovery Research, West Harwich, MA, USA; Abbott Nutrition, Analytical Research and Development, Columbus, OH, USA; Department of Obstetrics/Gynecology, Rowan University School of Osteopathic Medicine, Stratford, NJ, USA

**Keywords:** mother, infant, plasma, human, neonate, vitamin E, α-tocopherol, stereoisomer, natural, synthetic

## Abstract

**Background:**

α-Tocopherol (αT) is essential for fetal development. One study has shown that the human placenta preferentially transfers the natural stereoisomer, *RRR*-αT. But prenatal supplements generally contain synthetic αT (S-αT).

**Objectives:**

We aimed to determine if umbilical cord plasma is enriched for *RRR*-αT in racially diverse neonates from both uncomplicated and complicated pregnancies and if cord *RRR*-αT enrichment is impacted by maternal αT stereoisomer profile.

**Methods:**

We measured αT and αT stereoisomers in plasma from a randomly selected subset of 66 predominantly black and Hispanic maternal-fetal pairs from the Camden Study involving control (*n *= 28) and complicated pregnancies (*n *= 38). We collected maternal plasma at study entry (week 16 gestation; w16) and week 28 gestation (w28) and cord plasma at birth.

**Results:**

*RRR*-αT was the predominant stereoisomer in all maternal and cord plasma samples, but S-αT stereoisomers were found in most samples and comprised a high percentage of αT in some maternal-neonate pairs. Cord plasma had a higher percentage *RRR*-αT (*P *< 0.05) and lower percentage S-αT (*P *< 0.0001) than w28 plasma. Pregnancy status did not impact maternal or cord plasma concentrations of αT, *RRR*-αT, or S-αT; except plasma from complicated pregnancies was higher in S-αT at w28 than at w16 (*P *< 0.05). Maternal w28 αT did not correlate with cord αT. However, both maternal w28 αT and S-αT positively correlated with both cord S-αT (*r* = 0.340, *P *= 0.0049; *r* = 0.538, *P *< 0.00001) and percentage S-αT (*r* = 0.399, *P *= 0.001; *r* = 0.786, *P *< 0.00001) but negatively correlated with cord percentage *RRR*-αT (*r* = −0.399, *P *= 0.0009; *r* = −0.786, *P *< 0.00001).

**Conclusions:**

The proportion of *RRR*-αT was higher in cord compared with maternal plasma in both uncomplicated and complicated pregnancies. Our data suggest that maternal S-αT raises cord S-αT and decreases the proportion of *RRR*-αT in the neonatal circulation. Because the bioactivities of *RRR*-αT and S-αT differ, this warrants future research to determine the importance of our observations to neonatal αT status.

## Teaser

Natural vitamin E (VE) was enriched in infant's blood at birth but may be displaced by synthetic VE derived from mother's diet. Natural VE is more bioactive than synthetic VE.

## Introduction

α-Tocopherol (αT) is the only tocopherol structural isomer (α-, β- , δ-, and γ-tocopherol) that can meet vitamin E requirements (1). Supplementation studies in humans with vitamin E deficiency reveal that αT is critical to the function of the nervous system (2–4). Maternal αT insufficiency in early pregnancy leads to fetal resorption in rodents (5) and is associated with a higher risk of miscarriage in women (6).

The fetus acquires αT from maternal circulation via transplacental transport during gestation (7). It has been clearly established that cord plasma αT concentrations are significantly lower than, and generally do not correlate with, concentrations in matched maternal plasma (8–15). It remains unclear whether this reflects poor placental αT transport or other fetal or placental metabolic effects. Maternal supplements are routinely supplemented with αT to improve maternal status to ensure that the developing fetus has adequate access to αT for normal development.

αT used in maternal supplements can be isolated from plant oils like soy oil, or it can be chemically synthesized. The stereoisomeric composition of αT derived from these processes is different. αT isolated from plant oils exists as a single stereoisomer, *RRR*-αT (2*R*, 4′*R*, 8′*R*-αT), commonly referred to as natural vitamin E. In contrast, synthetic αT (*all rac*-αT; S-αT) is a racemic mixture of the 8 possible αT stereoisomers equally divided amongst 2*R* (*RRR, RRS, RSR, RSS*) and 2*S* (*SSS, SSR, SRS, SRR*) stereoisomers (1). S-αT has between 1.36- and 2-fold less vitamin E activity than an equal amount of *RRR*-αT (16).

Acuff et al. (17) found that when pregnant mothers were administered an equimolar mixture of *RRR*-αT and S-αT, the ratio of *RRR*-αT to S-αT in the fetal circulation was nearly double that of maternal circulation indicating that the fetal-placental unit selectively concentrated *RRR*-αT. This study was, however, small (*n* = 15 maternal-infant dyads) and individual αT stereoisomers were not analyzed. Because pregnant women are generally given supplements containing S-αT, 2 important questions were raised. First, is the preferential concentration of *RRR*-αT in umbilical cord plasma reported by Acuff et al. (17) also found in a larger, more diverse population with variable αT intake, potentially high maternal S-αT intake, and pregnancy complications? Secondly, in such a population, how much S-αT is found in umbilical cord plasma and is the concentration driven by the concentration of S-αT in maternal plasma? Our objective was to provide insight into these questions by analyzing individual αT stereoisomer concentrations in plasma from a subset of maternal-infant dyads from the Camden Study (18), a large, racially diverse population that included normal and high-risk pregnancies.

## Methods

### Study subjects

The current data were generated from a subset of 66 mother-infant pairs enrolled between January 1998 and April 2005 as part of the Camden Study (18), which was designed to study the effects of maternal nutrition in pregnant women from Camden, New Jersey, USA. The characteristics of 1231 pairs, which include the 66 pairs studied here, were previously provided in detail by Scholl et al. (19). The Institutional Review Board of the University of Medicine and Dentistry of New Jersey, School of Osteopathic Medicine (which later became Rowan University School of Osteopathic Medicine in 2013) approved the study procedures. Descriptive data for the 66 pairs studied here are presented in **Table 1** combined and by pregnancy status: control or complicated. The 66 pairs were selected because each had an available plasma sample for maternal weeks 16 (w16) and 28 (w28) of gestation and an umbilical cord plasma sample. Demographic information and maternal weight and height were obtained at w16 and w28 of gestation as previously described (19). Most of the study population was either black or Hispanic, with the combination accounting for ∼85% of the study population. Most of the mothers were single, experiencing their first pregnancy, were high school educated, did not smoke, and were not working. Pregnancy complications included preterm delivery (delivered <37 wk of gestation), small for gestational age (birth weight <10th percentile), low birth weight (birth weight <2500 g), and pre-eclampsia. Large for gestational age was defined by a birth weight >90th percentile of Zhang's standard that adjusts for maternal parity, ethnicity, and fetal sex (19), but was not categorized as a pregnancy complication. Information on current pregnancy outcome, complications, and infant abnormalities was abstracted from the prenatal record, the delivery record, delivery logbooks, and the infant's chart. Most of the mothers did not consume a maternal supplement at study w16, but the majority reported taking a maternal supplement at w28 of gestation. Supplemental αT intake was not different between mothers with control pregnancies and those with complicated pregnancies and changed from 4–5 mg/d at w16 to ∼14 mg/d during w28 of gestation. Infant sex and birth anthropometrics are presented in **Table 2** for the 66 pairs studied here. More than half the infants were female, and they were distributed evenly into the control and complicated pregnancies (Table 2). Gestation duration was based on the gravid woman's last normal menstrual period confirmed or modified by ultrasound scan. Gestational age averaged 38.5 wk and was similar for control and complicated pregnancies. However, birth weight was significantly lower for complicated pregnancies compared with control pregnancies. In contrast, birth length and head circumference were similar for control and complicated pregnancies.

**FIGURE 1:**
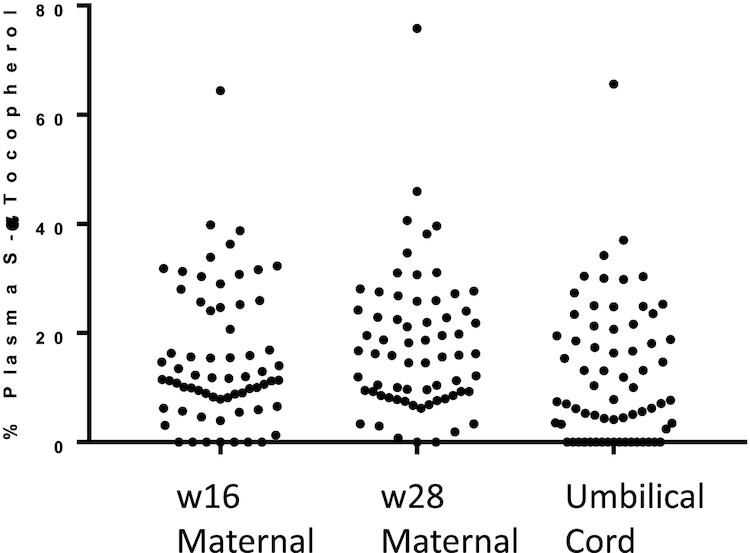
Percentage synthetic αT (S-αT) in week-16 (w16) and week-28 (w28) gestation maternal plasma and umbilical cord plasma from maternal-neonatal pairs (*n *= 66). S-*α*T, *RRS-* + *RSR- + RSS- +* 2*S-α*T.

**TABLE 1:** Maternal demographics and supplement use categorized by all, control, or complicated from 66 maternal-fetal pairs^1^

Criterion	All	Control	Complicated
*n*	66	28	38
Maternal ethnicity			
Black	31 (47)	16 (57)	15 (39)
Hispanic	25 (38)	8 (28)	17 (45)
Asian	1 (2)	1 (4)	0
White	9 (13)	3 (11)	6 (16)
Marital status			
Single	61 (92)	27 (96)	34 (90)
Married	5 (8)	1 (4)	4 (10)
First pregnancy	41 (62)	15 (54)	26 (68)
Maternal age, y	21.6 ± 5.61	21.9 ± 6.25	21.3 ± 4.62
	[12–40]	[15–40]	[12–31]
BMI, kg/m^2^	24.9 ± 5.84	24.9 ± 6.42	24.9 ± 5.50
	[16.6–45.1]	[16.6–45.1]	[17.9–42.1]
Maternal education			
Middle school	4 (6)	0	4 (10)
High school	52 (79)	25 (89)	27 (71)
GED	2 (3)	2 (7)	0
College	1 (2)	1 (4)	0
Unknown	7 (10)	0	7 (19)
Smoker?			
Yes	20 (30)	8 (29)	12 (32)
No	46 (70)	20 (71)	26 (68)
Working at w16?			
Part time	7 (11)	3 (11)	4 (10)
Full time	13 (19)	5 (18)	8 (22)
Maternal supplement use at w16: yes	19 (29)	6 (21)	13 (34)
Maternal supplement use at w28 gestation: yes	51 (77)	23 (82)	28 (74)
Supplemental αT intake, mg/d			
w16 gestation	5.06 ± 8.33	4.07 ± 7.94	5.78 ± 8.63
w28 gestation	13.9 ± 8.03	13.9 ± 7.58	13.9 ± 8.44

1 Values are *n* (%) or mean ± SD [range]. No statistical differences were detected between maternal demographic factors or supplement use for mothers with control or complicated pregnancies. GED, General Educational Equivalency – a high school equivalency diploma; w16, week 16 of gestation; w28, week 28 of gestation; αT, α-tocopherol.

**TABLE 2:** Birth status and anthropometrics for neonates from all, control, or complicated pregnancies from 66 maternal-fetal pairs^1^

Criterion	All	Control	Complicated
*n*	66	28	38
Female	38 (58)	18 (64)	20 (53)
Gestational age at w16 assessment, wk		17.0 ± 5.77	16.4 ± 5.68
Gestational age at w28 assessment, wk		29.9 ± 3.68	30.2 ± 3.21
Preterm delivery	17 (26)	0	17 (45)
Preterm delivery only		0	7
With low birth weight		0	6
With pre-eclampsia		0	1
With pre-eclampsia, LBW, small for GA		0	1
With large for gestational age		0	2
Small for gestational age	7 (11)	0	7 (18)
LBW with small for gestational age	2 (3)	0	2 (5)
Pre-eclampsia	10 (15)	0	10 (26)
Pre-eclampsia with large for gestational age	1 (2)	0	1 (3)
Gestational diabetes and pre-eclampsia	1 (2)	0	1 (3)
Large for gestational age	5 (8)	5 (18)	0
Infant birth weight, g	3137 ± 669	3465 ± 469^a^	2895 ± 669^b^
	[1478–5354]	[2790–4300]	[1478–5354]
Infant birth length, cm	50.0 ± 3.11	50.8 ± 3.09	49.5 ± 3.04
	[41.5–57.0]	[44.0–57.0]	[41.5–56.0]
Infant birth head circumference, cm	33.3 ± 1.98	33.9 ± 1.76	32.6 ± 2.10
	[27.5–38.5]	[30.0–37.0]	[27.5–38.5]

1 Values are presented as *n* (%), or as mean ± SD [range]. ^a, b^Labeled means in a row without a common lowercase superscript letter differ (*P *< 0.0001) by 1-factor ANOVA and Tukey post hoc test. LBW, low birth weight; w16, week 16 of gestation; w28, week 28 of gestation.

Samples of the mother's blood were obtained at w16 of the study and at w28 gestation, aliquoted, and stored at −80°C until analysis.

### Analysis of αT and its stereoisomers

αT and its stereoisomers were assessed as we described previously (20). In brief, samples were extracted with hexane following saponification as described (21). A portion of the hexane was dried and reconstituted to measure αT by HPLC with electrochemical chemical detection (ECD) as described (20). αT was quantified at the dominant oxidation potential relative to an external αT standard (Sigma) that was validated against certified reference material (National Institute of Standards and Technology SRM 968f). To assess αT stereoisomers, the remaining portion of the aforementioned hexane extract was used to measure the percentage distribution of αT stereoisomers as we described (20). In brief, the hexane extract was dried under nitrogen gas, resolubilized, and the reconstituted sample was methylated under basic conditions prior to extracting with hexane. Samples were then separated and detected by HPLC with fluorescence detection using a chiral separation column and excitation/emission settings of (290_nm_/330_nm_). Under these conditions, each specific 2*R* stereoisomer of αT (*RRR*-, *RRS*-, *RSR*-, and *RSS*-αT) was determined along with a single peak for total 2*S* stereoisomers. Peak area of each stereoisomer was calculated to determine percentage distribution, and their molar concentration determined based on the concentration of total α-T obtained by HPLC-ECD. The detection limit was ∼0.02 μmol/L for *S* stereoisomers and 0.05 μmol/L for *R* stereoisomers.

### Statistics

All data were analyzed with GraphPad Prism version 5.04 for Windows (GraphPad Software; www.graphpad.com). Normality and homogeneity of variance were evaluated using the D'Agostino–Pearson test using a cutoff of −2 and 2 before further testing. Prior to comparison, data that were not normal were log transformed (Y + 1) if zero was a value, or log transformed (Y) if not. Comparisons of birth anthropometrics between control and complicated pregnancies and maternal w16 plasma, maternal w28 plasma, and cord plasma αT values were done using 1-factor ANOVA, and when appropriate, differences between means were evaluated using Tukey multiple comparison test. Means that differed significantly following Tukey multiple comparison test are denoted using unique letter subscripts (*P* < 0.05). Pearson correlation test was used to test for significant correlations. A false discovery rate analysis using a 1% rate was used to adjust for multiple comparisons within correlation matrices. Unless indicated otherwise, data are expressed as means ± SD of the mean.

## Results

Total αT (*P *< 0.05) and S-αT stereoisomers (*P *< 0.05) in maternal plasma were higher at w28 of gestation than at w16, but *RRR*-αT was not (**Table 3**). Maternal plasma had a >4-fold higher mean αT concentration than cord plasma (*P *< 0.0001) (Table 3). Scatterplots of total αT values are provided in **Supplemental Figure 1**. Maternal plasma also had a ∼4-fold higher concentration of *RRR*-αT (*P *< 0.0001) and a 6-fold higher concentration of S-αT (*P *< 0.0001) compared with cord plasma. *RRR*-αT was the most common αT stereoisomer in all maternal and cord samples. At w16, 8/66 maternal plasma samples contained only *RRR*-αT, but by w28 this was true of only 2/66 mothers. At birth, 15/66 cord plasma samples contained only *RRR*-αT; however, 38 of the remaining 51 neonates had each S-αT stereoisomer in their plasma. The individual S-αT stereoisomers in maternal and umbilical cord plasma are provided in **Supplemental Table 1**.

**TABLE 3:** α-Tocopherol, *RRR*-α-tocopherol, and synthetic α-tocopherol stereoisomer concentration and proportions in maternal plasma and umbilical cord plasma^1^

	Maternal week 16, μmol/L	Maternal week 16, % αT	Maternal week 28, μmol/L	Maternal week 28, % αT	Umbilical cord, μmol/L	Umbilical cord, % αT
αT	23.6 ± 6.89^b^	—	29.8 ± 7.67^a^	—	6.39 ± 3.15^c^	—
	[13.2–47.5]		[18.3–66.0]		[1.97–21.5]	
*RRR*-αT	19.6 ± 5.29^a^	85.6 ± 11.6^ab^	23.9 ± 5.12^a^	82.0 ± 12.7^b^	5.52 ± 2.76^c^	87.8 ± 12.5^a^
	[14.9–35.5]	[37.7–100]	[14.9–35.5]	[24.2–100]	[1.91–18.9]	[34.3–100]
Synthetic αT	4.04 ± 4.10^b^	14.4 ± 11.2^a^	5.98 ± 6.79^a^	18.0 ± 12.7^a^	0.871 ± 1.21^c^	12.2 ± 12.5^c^
	[0–22.7]	[0–66.3]	[0–50.1]	[0–75.8]	[0–8.25]	[0–65.7]

1 Values are mean ± SD [range]; *n *= 66. Maternal plasma from week 16 or week 28 of gestation. Umbilical cord plasma collected at birth. Synthetic αT: *RRS *+ *RSR *+ *RSS *+ 2*S* stereoisomers. ^a–c^ Labeled means within a row for μmol αT/L or % αT without a common lowercase superscript letter differ: ^a,c; b,c^*P *< 0.0001; ^a,b^*P *< 0.05 by 1-factor ANOVA and Tukey post hoc test. Data were normalized using log(y). αT, α-tocopherol.

Umbilical cord plasma had a higher mean percentage *RRR*-αT than did maternal w28 plasma, but not w16 maternal plasma (Table 3). Percentage *RRR*-αT ranged from a minimum of 24% and 34% in maternal and cord plasma, respectively, to a maximum of 100% in both. Umbilical cord plasma had a lower mean percentage S-αT than maternal plasma at either w16 or w28. The percentage S-αT ranged from 0% to as high as 76% and 66% in maternal and cord plasma, respectively. Individual data for plasma percentage S-αT are provided in **Figure 1** and reveal that the majority of maternal and umbilical cord samples contained S-αT. Approximately one-quarter to one-third of plasma samples had >20% S-αT, an arbitrary cutoff value for illustrative purposes. Consistent with these observations, umbilical cord plasma had a higher mean *RRR*-αT/S-αT ratio (9.59 ± 8.86; range: 0.523–40.2) than w28 maternal plasma (6.63 ± 5.22; range: 0.368–26.0) (*P *< 0.001).

Control and complicated pregnancies were not different for maternal plasma αT, *RRR*-αT, or S-αT concentrations (**Table 4**). Whereas maternal values generally did not change from w16 to w28, mean S-αT concentration for w28 complicated pregnancies was higher than complicated pregnancy w16 S-αT (*P *< 0.05). There were no differences in αT, *RRR*-αT, or S-αT concentrations in cord plasma from control or complicated pregnancies (Table 4).

**TABLE 4:** α-Tocopherol, *RRR*-α-tocopherol, and synthetic α-tocopherol stereoisomer concentration in maternal and umbilical cord plasma from control and complicated pregnancies^1^

						Umbilical cord plasma complicated pregnancies
	Maternal plasma control pregnancies		Maternal plasma complicated pregnancies		Umbilical cord plasma control pregnancies	
	Week 16	Week 28	Week 16	Week 28		
αT, μmol/L	22.3 ± 5.86	28.1 ± 5.39	24.5 ± 7.50	30.5 ± 9.21	6.07 ± 2.63	6.56 ± 3.47
	[13.2–36.7]	[18.3–39.4]	[13.8–47.5]	[13.1–66.0]	[2.79–14.0]	[1.97–21.5]
*RRR*-αT, μmol/L	18.7 ± 4.03	24.2 ± 5.62	20.2 ± 5.70	23.4 ± 5.03	5.38 ± 2.63	5.59 ± 2.85
	[11.3–28.3]	[14.9–35.5]	[11.3–35.4]	[12.7–33.1]	[2.65–14.0]	[1.91–18.9]
Synthetic αT, μmol/L	3.57 ± 3.35^b^	3.97 ± 3.07^ab^	4.28 ± 4.60^b^	7.13 ± 8.27^a^	0.695 ± 0.735	0.975 ± 1.44
	[0–13.7]	[0–13.6]	[0–22.7]	[0–50.0]	[0–2.79]	[0–8.25]

1 Values are mean ± SD [range]. *n* = 66; control pregnancies, *n *= 28; complicated pregnancies, *n *= 38. Umbilical cord plasma collected at birth. Synthetic αT: *RRS *+ *RSR *+ *RSS *+ 2*S* stereoisomers. Data were normalized by log (y) transformation prior to 1-factor ANOVA analyses. ^ab^ Labeled means in a row for maternal or umbilical cord without a common lowercase superscript letter differ (*P *< 0.05) by 1-factor ANOVA and Tukey post hoc test. Week 16, week 16 of gestation; Week 28, week 28 of gestation; αT, α-tocopherol.

Maternal w28 plasma total αT concentration did not correlate with cord plasma total αT or *RRR*-αT concentration, but did correlate positively with both cord plasma S-αT concentration and percentage S-αT (**Table 5**). Maternal plasma total αT concentration negatively correlated with cord plasma percentage *RRR*-αT. In contrast, the concentration of *RRR*-αT in maternal w28 plasma was not related to the concentration of αT, *RRR*-αT, or S-αT in cord plasma. Maternal w28 plasma S-αT concentration correlated positively with both cord plasma S-αT and percentage S-αT, and negatively with cord percentage *RRR*-αT. Maternal w28 plasma αT and S-αT correlations with individual S-αT stereoisomers in umbilical cord are shown in **Supplemental Table 2**.

**TABLE 5:** Relation between maternal and umbilical cord plasma α-tocopherol stereoisomer concentrations and proportions^1^

Maternal plasma	Statistic	Umbilical cord plasma αT, μmol/L	Umbilical cord plasma *RRR*-αT, μmol/L	Umbilical cord plasma synthetic αT, μmol/L	Umbilical cord plasma *RRR*-αT, % αT	Umbilical cord plasma synthetic αT, % αT
αT, μmol/L	*r*			0.340	−0.399	0.399
	*P*			0.0049	0.0009	0.001
*RRR*-αT, μmol/L	*r*					
	*P*					
Synthetic αT, μmol/L	*r*			0.538	−0.786	0.786
	*P*			<0.00001	<0.00001	<0.00001

1 Values are Pearson correlation coefficients, *r*, and *P* values. *n *= 66. Maternal plasma was from week 28 of gestation and umbilical cord plasma was collected at birth. Synthetic αT: *RRS *+ *RSR *+ *RSS *+ 2*S* stereoisomers. Data were normalized by log (y + 1) transformation prior to correlation analyses. A false discovery rate threshold of 1% was used to correct for multiple analyses. Empty cells indicate no significant correlation, αT, α-tocopherol.

Mean maternal supplemental intake of αT positively correlated with maternal S-αT at both w16 (*r* = 0.251, *P *< 0.05) and maternal w28 (*r* = 0.304, *P *< 0.02), but did not correlate with maternal αT or *RRR*-αT, or with umbilical cord αT measures. In control pregnancies, supplemental αT intake was not correlated with maternal plasma αT. In contrast, in complicated pregnancies, mean maternal supplemental intake of αT positively correlated with w28 αT (*r* = 0.397, *P *< 0.02), and with maternal S-αT at both w16 (*r* = 0.367, *P *< 0.02) and w28 (*r* = 0.416, *P *< 0.01).

## Discussion

We measured the αT stereoisomer profile in maternal plasma and matched umbilical cord plasma in a cohort that was racially diverse and included both complicated and uncomplicated pregnancies. We found that *RRR*-αT was the most common αT stereoisomer in all maternal and umbilical cord plasma samples analyzed, and that the proportion of *RRR*-αT was higher in cord plasma than in w28 maternal plasma. This latter finding is consistent with that of Acuff et al. (17), who found in a small study involving deuterated αT that the fetal placental unit concentrated *RRR*-αT compared with maternal plasma. Because our study population was heavily weighted with minority and low socio-economic/poorly educated mothers together with complicated pregnancies, our findings suggest that the enrichment of fetal circulation with *RRR*-αT is a general phenomenon in humans. This is likely explained by the presence of α-TPP at the placental interface between the maternal and fetal circulation (22). α-TPP preferentially binds *RRR*-αT compared with other stereoisomers of αT (16).

Our study population included pregnancies with and without complications. Complications included preterm delivery, small for gestational age, pre-eclampsia, and gestational diabetes. Pregnancy status had no effect on maternal and umbilical cord plasma αT measures except that maternal w28 plasma samples had a higher concentration of S-αT. We cannot explain this difference, but speculate it might reflect differential adherence to prenatal supplement use.

Despite the predominance of *RRR*-αT in all plasma samples studied, we also generally found measurable and variable concentrations of S-αT in plasma. More than three-quarters of both maternal and cord samples contained measurable concentrations of S-αT. Most maternal and umbilical cord plasma samples had between 10% and 40% of αT as S-αT. One maternal-neonate pair had more than two-thirds S-αT, which might be explained by a specific nucleotide polymorphism previously reported for α-tocopherol transfer protein (αTTP) (23). Prenatal maternal supplements in the United States generally contain S-αT and generally provide a higher daily intake of αT than diet. Indeed, maternal plasma concentrations of αT increased concomitant with increased reports of supplement use. The increase in maternal plasma αT appeared to have been driven by increased concentrations of plasma S-αT. Therefore, we consider it likely that prenatal vitamin consumption explains the widespread presence of S-αT in the plasma samples studied. Consistent with this, maternal plasma αT and S-αT concentrations were positively correlated with S-αT in umbilical cord plasma. This suggests that despite the presence of αTTP in the placenta, a significant proportion of the fetal-placental units studied could not fully compensate for maternal plasma S-αT concentrations. This in turn led to lower concentrations of *RRR*-αT based on the negative correlation found between maternal plasma S-αT and umbilical cord *RRR*-αT. Taken together, this suggests that increasing maternal αT status through prenatal supplement use is likely to be associated with a decrease in umbilical cord plasma *RRR*-αT despite a selective placental bias for *RRR*-αT.

Our data are not entirely consistent with the interpretation by Acuff et al. (17) of their findings. They speculated that their results were numerically consistent with the selective transport of *RRR*-αT and 1 S-αT stereoisomer by the fetal-placental unit. Here, 35 of the 66 cord plasma samples studied had >1 S-αT stereoisomer. Our data are consistent with increased tissue proportions of S-αT stereoisomers in animals that received doses of S-αT that exceeded the dose of *RRR*-αT (24–26).

Consistent with previous reports, we found that mean maternal plasma αT concentration was markedly higher than that of cord plasma (8–13, 15). In addition, the maternal plasma αT concentrations reported here during pregnancy are similar to values reported by others during pregnancy, including the complete cohort by Scholl et al. (19) and elsewhere (8–9, 11–15), but are higher than those reported by Kiely et al. (10). In addition, the αT concentrations reported here are similar to those reported for healthy nonpregnant women (27–29) indicating that the maternal concentrations were not unusually high or low. The few women at the lower end of the range in this study approached the median maternal plasma αT concentration that Shamim et al. (6) found positively related with elevated risk of miscarriage. The cord plasma αT concentrations reported here, including those from complicated pregnancies, are directionally higher than those reported from a large cohort—4.3 μmol αT/L (15)—and a smaller cohort reporting 3.6 μmol αT/L (13), but are similar to those reported for smaller cohorts with mean values ranging from 5.5 to 7.7 μmol/L (8–12). Thus, the αT concentrations reported here are in line with those of previous reports, despite the poor economic and education status of the current cohort.

A limitation of this study is that maternal plasma was not collected at delivery. A second limitation is that the sample size did not allow for incorporation of potential confounders such as maternal age, gestational length, neonate sex, and pregnancy status into our statistical analyses. Strengths of the study include the heavy representation of black and Hispanic maternal-neonate pairs, the inclusion of complicated pregnancies, and the direct quantitation of αT stereoisomers.

In conclusion, we found that *RRR*-αT was the most common αT stereoisomer in all maternal and umbilical cord plasma samples, and that umbilical cord plasma contained a higher proportion of *RRR*-αT than maternal plasma. Nonetheless, most samples had measurable concentrations of S-αT, which could account for a substantial percentage of total αT. These observations were not influenced by pregnancy status. Maternal plasma concentrations of αT and S-αT were positively correlated with umbilical cord plasma S-αT, but negatively correlated with cord *RRR*-αT. We believe these finding to be important for several reasons beyond the well-documented differences in vitamin E activity between *RRR*-αT and S-αT (5). αT has increasingly been found to influence gene expression (30–35), and more recently, evidence is accumulating that S-αT stereoisomers and *RRR*-αT have differential impacts on gene expression in the brain (20, 36, 37). In addition, improved maternal αT status appears to positively influence fetal growth (19, 38–42), but recently cord plasma αT concentration was reported to be negatively related to neonate 5-min Apgar (physician ratings of the newborn infant's appearance, pulse, grimace, activity, respiration) (38). Because umbilical cord proportions of *RRR*-αT and S-αT were related to maternal plasma αT measures, we believe future research is warranted to explore the relative impact of *RRR*-αT and S-αT on fetal and neonatal health.

## Supplementary Material

nzab073_Supplemental_Files

## Notes

Supported by Abbott Nutrition and R01MD007828.

Author disclosures: MJK and KJS are employed by Abbott Nutrition. All other authors report no conflicts of interest

Supplemental Figure 1 and Supplemental Tables 1 and 2 are available from the “Supplementary data” link in the online posting of the article and from the same link in the online table of contents at https://academic.oup.com/cdn/.

Abbreviations used: ECD, electrochemical detection; S-αT, synthetic α-tocopherol; w16, week 16; w28, week 28; αT, α-tocopherol; αTTP, α-tocopherol transfer protein; 2*R, RRR + RRS + RSR + RSS*-; 2*S*, *SSS + SSR + SRS + SRR*-.
