# Supplementary material for: α-Tocopherol Stereoisomer Profiles in Matched Human Maternal and Umbilical Cord Plasma
Source: Curr Dev Nutr. 2021 May 3;5(6):nzab073. doi: 10.1093/cdn/nzab073 (PMC8178107; doi:10.1093/cdn/nzab073)
Supplement: nzab073_Supplemental_Files [file nzab073_supplemental_files.zip › Supplemental_Tables_1_and_2_11.3.20.docx]

**SUPPLEMENTAL Table 1**. Synthetic α-tocopherol stereoisomer concentration and proportions in maternal plasma and infant cord plasma^1^

|  | αT (umol/L) | | | | Percent Total αT | | | |
| --- | --- | --- | --- | --- | --- | --- | --- | --- |
|  | Maternal | All Cord | Control Cord | Complicated Cord | Maternal | All Cord | Control Cord | Complicated Cord |
| Synthetic  αT^2^ | 5.98+6.79^a^  (0, 50.1) | 0.871+1.21^b^  (0, 8.25) | 0.695+0.735  (0, 2.79) | 0.975+1.44  (0, 8.25) | 18.0 + 12.7^a^  (0, 75.8) | 12.2+12.5^b^  (0, 65.7) | 11.9+11.2  (0, 36.9) | 12.+ 13.5  (0, 65.7) |
| *RRS*-αT | 1.68+1.91^c^  (0, 12.7) | 0.215+0.375^d^  (0, 2.42) | 0.163+0.259  (0, 0.906) | 0.252+0.439  (0, 2.42) | 5.10 + 4.07^a^  (0, 19.2) | 3.06+4.53^b^  (0, 19.2) | 2.73+4.12  (0, 14.5) | 3.31+4.84  (0, 19.2) |
| *RSR*-αT | 2.53+2.02^c^  (0, 14.4) | 0.309+0.476^d^  (0, 2.68) | 0.237+0.320  (0, 1.13) | 0.360+0.561  (0, 2.68) | 8.00 + 4.09^a^  (0, 21.8) | 4.07+4.84^b^  (0, 21.3) | 3.89+4.95  (0, 13.1) | 4.21+4.83  (0, 21.3) |
| *RSS*-αT | 1.17+1.62 ^c^  (0, 10.9) | 0.126+0.274^d^  (0, 1.89) | 0.114+0.208  (0, 0.929) | 0.134+0.317  (0, 1.89) | 3.53 + 3.29^a^  (0, 16.6) | 1.73+3.05^b^  (0, 15.1) | 1.86+3.25  (0, 14.5) | 1.632.94  (0, 15.1) |
| 2*S* | 0.64+1.48^c^  (0, 12.1) | 0.209+0.210^d^  (0, 1.859) | 0.184+0.152  (0, 0.658) | 0.229+0.243  (0, 1.26) | 1.80 + 2.39^d^  (0, 18.3) | 3.36+2.85^c^  (0, 14.9) | 3.39+3.25  (0, 14.9) | 3.34+2.82  (0, 10.0) |

^1^ Values are mean + standard deviation (range). n=66. Maternal plasma from week 28 of gestation. Umbilical cord plasma was collected at birth from Control (n=28) or Complicated pregnancies (n=38). Synthetic αT: *RRS*+*RSR*+*RSS*+*2S* stereoisomers. Data were transformed using log(y). ^a-d^ Labeled means in a row for αT or Percent Total αT without a common lowercase superscript letter differ, ^a,b^ *P*<0.0001; ^c,d^ *P*<0.05 by one-way ANOVA and Tukey’s post hoc test.

**SUPPLEMENTAL Table 2.** Relationship between maternal plasma α-tocopherol stereoisomer concentrations and proportions and the cord plasma concentrations and proportions of individual synthetic α-tocopherol stereoisomers ^1^

|  |  |  | Cord (μmol/L) | | | | Cord (%) | | | |
| --- | --- | --- | --- | --- | --- | --- | --- | --- | --- | --- |
| Maternal |  |  | *RRS*-αT | *RSR*-αT | *RSS*-αT | 2*S* | *RRS*-αT | *RSR*-αT | *RSS*-αT | 2*S* |
| αT | μmol/L | r  *P* | 0.347  0.0040 |  | 0.321  0.0080 | 0.372  0.002 | 0.309  0.0110 |  |  |  |
| *RRR*-αT | μmol/L | r  *P* |  |  |  |  |  |  |  |  |
| Synthetic αT^3^ | μmol/L | r  *P* | 0.583  <0.0001 | 0.426  0.0003 | 0.502  0.0002 | 0.415  0.0005 | 0.603  <0.0001 | 0.500  0.0002 | 0.502  0.0002 |  |

^1^ Values are Pearson Correlation coefficients, r, and *P* values. n=66. Data were normalized by log (y+1) transformation prior to correlation analyses. A False Discovery Rate threshold of 1% was used to correct for multiple analyses. Empty cells indicate no significant correlation. Maternal plasma was from week 28 of gestation and umbilical cord plasma was collected at birth. Synthetic αT: *RRS*+*RSR*+*RSS*+*2S* stereoisomers.
