# Supplementary material for: α-Tocopherol Stereoisomer Profiles in Matched Human Maternal and Umbilical Cord Plasma
Source: Curr Dev Nutr. 2021 May 3;5(6):nzab073. doi: 10.1093/cdn/nzab073 (PMC8178107; doi:10.1093/cdn/nzab073)
Supplement: nzab073_Supplemental_Files [file nzab073_supplemental_files.zip › Supplemental_Figure_1.pptx]

## Slide 1
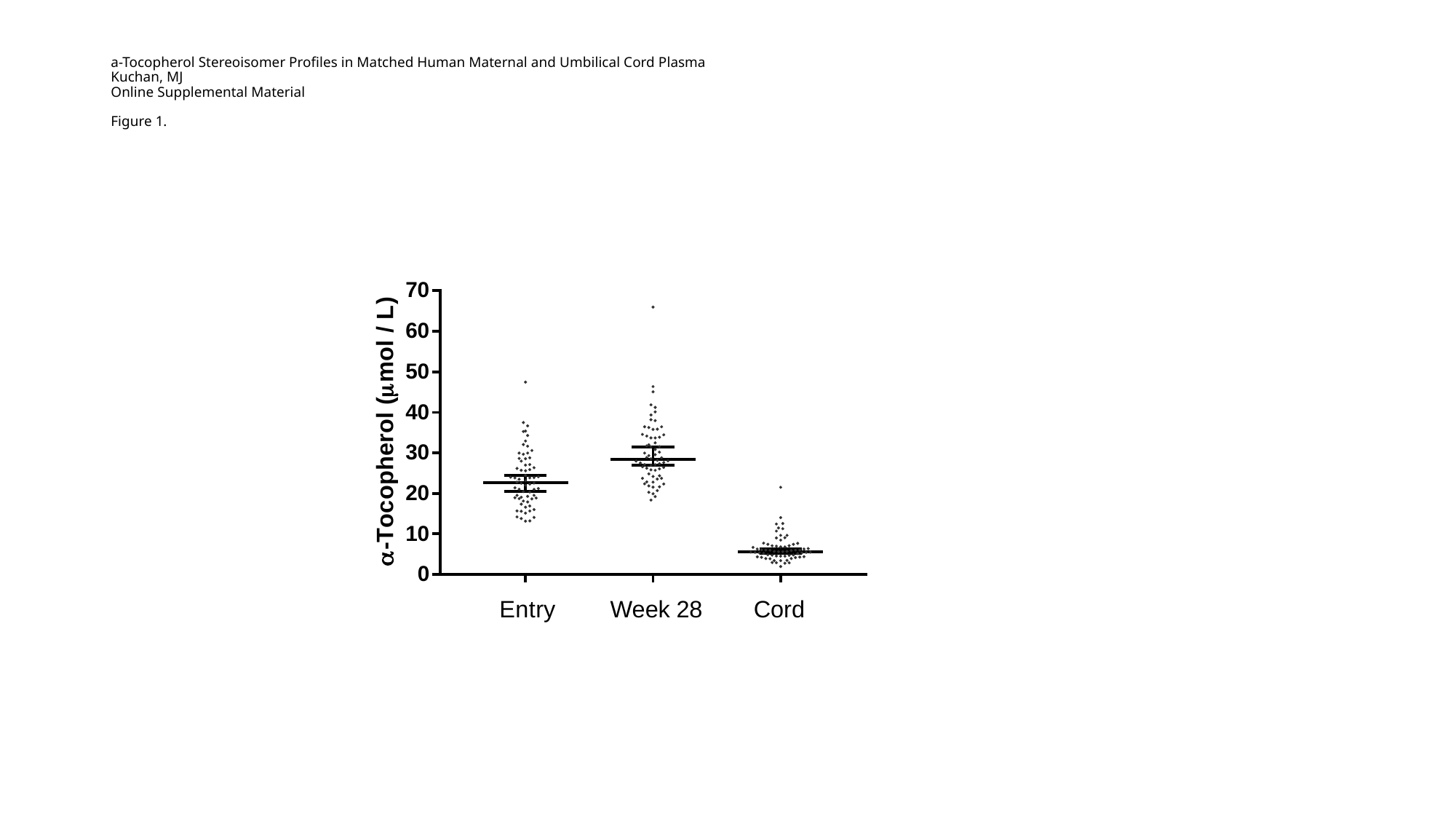

# a-Tocopherol Stereoisomer Profiles in Matched Human Maternal and Umbilical Cord Plasma Kuchan, MJ Online Supplemental Material Figure 1.
